# Analysing the use of the Australian Health Star Rating system by level of food processing

**DOI:** 10.1186/s12966-018-0760-7

**Published:** 2018-12-13

**Authors:** Sarah Dickie, Julie L. Woods, Mark Lawrence

**Affiliations:** School of Exercise and Nutrition Sciences, Institute for Physical Activity and Nutrition (IPAN), Deakin University, 221 Burwood Highway, Burwood, VIC 3125 Australia

**Keywords:** Ultra-processed food, Front-of-pack labelling, Food processing, NOVA, Health star rating, Nutrient profiling, Dietary guidelines, Food reformulation, Behavioural nutrition

## Abstract

**Background:**

The consumption of ultra-processed foods is associated with diminished dietary quality and adverse health outcomes. The Australian Health Star Rating (HSR) is a nutrient-based front-of-pack (FOP) labelling system that assesses the ‘healthiness’ of foods on a scale of 0.5 to 5 stars based on their content of ‘risk’ and ‘positive’ nutrients. This study aimed to analyse the use of health stars on new packaged food products entering the Australian marketplace by level of food processing.

**Methods:**

The Mintel Global New Product Database (GNPD) was searched to identify the number of stars displayed on the labels of all new packaged food products participating in the HSR system released into the Australian retail food supply between 27 June 2014 (the endorsement date) and 30 June 2017. Products were categorised by the four NOVA food processing categories: unprocessed and minimally processed (MP), processed culinary ingredients (PCI), processed (P), and ultra-processed (UP), and the distribution of the star ratings within each category was compared and analysed.

**Results:**

The majority of new food products displaying an HSR were UP (74.4%), followed by MP (12.5%), P (11.6%), and PCI (1.5%). The median HSR of MP products (4.5) was significantly higher than the median of P (4) and UP products (3.5) (all *p* < 0.05). In all NOVA categories HSR profiles were distributed towards higher star ratings, and the majority (77%) of UP products displayed an HSR ≥ 2.5.

**Conclusions:**

The HSR is being displayed on a substantial proportion of newly released UP foods. Technical weaknesses, design flaws and governance limitations with the HSR system are resulting in 3 out of 4 instances of these UP foods displaying at least 2.5 so-called ‘health’ stars. These findings add further evidence to concerns that the HSR system, in its current form, is misrepresenting the healthiness of new packaged food products and creating a risk for behavioural nutrition.

**Electronic supplementary material:**

The online version of this article (10.1186/s12966-018-0760-7) contains supplementary material, which is available to authorized users.

## Background

Dietary risk factors are a leading contributor to the burden of disease in Australia, implicated in the cause of cardiovascular disease, diabetes and certain cancers [1]. The presence of a high proportion of ultra-processed (UP) foods, a term coined by researchers in Brazil to refer to industrially-formulated foods that contain few whole food components [2], has been identified as a characteristic of dietary patterns associated with a number of these risk factors. Health associations with the consumption of a high proportion UP foods can be attributed directly to them being vectors for delivering high amounts of salt, sugar and fats, and indirectly to the displacement of healthy dietary patterns consisting of unprocessed and minimally processed (MP) foods. Also, independent of their poor nutrient composition, mechanistic research has identified physical and chemical changes to the original whole food as plausible explanations of the relationships between UP foods and health outcomes [3]. The relationship between the consumption of UP foods and impaired diet quality has been reported in Canada [4], Brazil [5], Columbia [6], the United States [7], and Europe [8]. Research has also shown a positive association between higher intake of UP foods and disease risk factors, including higher BMI [9], metabolic syndrome in adolescents [10], altered lipid profiles in children [11], hypertension in adults [12], and overall cancer and breast cancer risk [13]. Additionally, UP foods are associated with negative environmental impacts. The added post-production processing steps required for their preparation typically have a higher: contribution to avoidable greenhouse gas emissions; use of finite natural resources; and pressures on biodiversity, relative to less processed foods [14, 15].

The NOVA system is a relatively new and increasingly used approach to classify the ‘healthiness’ of foods. The classification is based on the nature, extent, and purpose of a food’s industrial processing to help understand links between dietary quality and health outcomes [16]. It has been applied extensively in the assessment of dietary patterns [2] and forms the basis of the Brazilian and Uruguayan dietary guidelines recommendations, which include the avoidance of UP products [17, 18]. An increase in the availability, purchasing and consumption of UP foods over time has been observed in Brazil [19], Sweden [20], Norway [21], and Australia [22]. Three studies have investigated the level of food processing in the Australian food supply, finding more than one third of commonly consumed foods are UP products [23], 95% of high market share UP products contain added sugar [24], and 82% of all new packaged products released in 2015 were UP [25]. In New Zealand, a country that shares a common food regulation system with Australia, it has been reported that UP products are the most available packaged products in supermarkets [26].

The World Health Organization (WHO) recommends implementing interpretive front-of-pack (FOP) labelling as part of comprehensive programmes to address childhood obesity [27]. The Codex Alimentarius Commission (Codex) is also in the process of creating guidelines to ensure global consistency of FOP labels [28]. Although many different types of FOP labelling systems have emerged around the world, there is no international consensus about which nutrients (or level of food processing) or cut-off levels should be included in models underpinning them, in order to maximize the likelihood of promoting healthier diets. In 2011, an Australian government commissioned review of food labelling recommended a single standardised interpretive FOP label that should indicate a food’s healthiness in respect to the Australian Dietary Guidelines (ADGs) [29].

In response to recommendations, in June 2014, the Australian Government introduced the Health Star Rating (HSR) system, a voluntary interpretive FOP label aimed at improving the population’s ability to make healthier food choices [30]. A complementary objective of the HSR system as part of its role within the Australian Government’s ‘Healthy Food Partnership’ is to provide an incentive to encourage manufacturers to formulate new food products entering the Australian marketplace [31]. In contrast to the food-oriented NOVA classification system, the HSR system is nutrient-oriented, rating the healthiness of a food using a nutrient profiling procedure. An algorithm calculates a score based on a food’s nutrient composition, which is then converted to a ‘health’ rating ranging from 0.5 to 5 stars – “*the more stars, the healthier*” [30, 32]. Baseline points are calculated for the ‘risk’ components of energy, saturated fat, sodium and total sugars; and modifying points for ‘positive’ components, the proportion of fruit, nut, vegetable and legume (FVNL), fibre, and protein content [33]. The HSR system has had a modest implementation in Australia with approximately 7000 products displaying the label [34] and of the 12,108 new products released between 27 June 2014 and 30 June 2017, 1269 displayed a HSR, representing approximately 18% of all products carrying an HSR [35].

The HSR system is a controversial approach for promoting healthy food selection. Previously we have reported that 57% of new discretionary (mostly energy-dense, nutrient-poor) food products displaying an HSR are doing so with a rating of ≥2.5 health stars, effectively undermining the recommendations of the ADGs [35]. Others have assessed that there is ‘good’ (86.6%) alignment between the HSR and ADGs [36]. Though this assessment used a cut-off of ≥3.5 stars, which assumes that displaying up to 3 health stars on discretionary foods is consistent with ADG recommendations despite the ADGs advising “they are not an essential or necessary part of health dietary patterns” and most Australians consume too many of them [37]. Although a ≥ 3.5 cut-off has been used in several studies to date [36, 38–40], there is no evidence this cut-off is predictive of health outcomes, nor is there a formal convention for its adoption. Previously we have challenged the appropriateness of assessing discretionary foods that display 3 (out of 5) health stars, i.e. a ‘pass’ level, as being consistent with ADG recommendations [41].

Whereas the ADG recommendations were informed by evidence synthesised from food/dietary pattern and health outcome relationships, the HSR design calculates scores on a limited selection of nutrients and arbitrary cut off levels informed by expert opinion [42]. Consistent with all other nutrient profiling-informed FOP labelling systems, the HSR system lacks evidence that it is predictive of health outcomes and the system’s implementation is yet to demonstrate effectiveness in promoting healthier diets.

Apart from two small studies suggesting the HSR system can misleadingly allocate high HSRs to UP products [24, 43], no studies have comprehensively assessed new food products entering the marketplace displaying the HSR label against a food processing classification scheme. This study aimed to analyse the use of health stars on new packaged food products entering the Australian marketplace by level of food processing to answer the research question: ‘Is the Health Star Rating system misrepresenting the healthiness of new food products?’. The study objectives were to: i) Describe the HSR profile of new food products by level of food processing; and ii) Examine the characteristics of MP, P, and UP foods displaying HSRs by comparing within ADG food groups.

## Methods

### Data collection

Systematic sampling of all new Australian food and beverage product launches displaying a HSR using the Mintel Global New Products Database (GNPD) between 27/6/14 (the date that the Australia and New Zealand Ministerial Forum on Food Regulation endorsed the HSR system [44]) and 30/6/17 was conducted. Mintel GNDP is an industry resource that collects detailed information on new and updated food and beverage products released on to the market worldwide. Mintel GNDP shoppers are trained to find new products when packaging indicates the product is: a re-launch, new formulation, new product, and/or new variety/extension; and/or new packaging can be recognised from the average shoppers’ perspective. One researcher (SD) visually examined all food product labels displayed in the database to check for the presence of an HSR. Detailed information on all products was extracted, including the number of health stars displayed, GNPD food category and sub-category, release date, product description, packaging images, nutrition composition, and ingredients list.

### Data analysis

NOVA categorises foods into four groups: unprocessed or minimally processed (MP) foods (e.g. eggs, fresh fruit and vegetables, unsweetened juice, fresh or frozen meats, dried pulses, flakes or flours made from grains, pasteurised milk, pasta, and teas); processed culinary ingredients (PCI) (e.g. plant oils, animal fats, vinegars, sugars, and salt); processed (P) foods (recognisable versions of original foods manufactured by adding salt or sugar; e.g. canned vegetables, cured meats, and unpackaged freshly-baked breads); and ultra-processed (UP) foods (unrecognisable food-like formulations, containing preservatives and additives such as stabilisers, emulsifiers, sweeteners, colours, and flavours) [2].

The NOVA classification system previously has been applied to items in the AUSNUT 2011–2013 food composition database [23], which reflects the Australian food supply during this time period [45]. O’Halloran et al. classified all items in AUSNUT based on the four NOVA categories: MP, PCI, P, and UP [23]. The supplementary information provided with the O’Halloran et al. publication listing all items in AUSNUT 2011–13 with assigned coding formed the primary classification method for the current study. Where the AUSNUT 2011–2013 did not provide suitable descriptions for products in the sample, items were classified by referring to the NOVA categories described by Monteiro et al. [46]. A second researcher (JW) conducted quality control using previously published methods [47, 48]. This involved checking coding decisions on a 5% sample of the data, and if disagreement occurred, consensus was reached by discussion among the three primary researchers.

### Statistical analysis

All statistical analyses were conducted in IBM SPSS Statistics version 23. Descriptive statistics, including median, range and interquartile range (IQR) of HSR scores were produced for the total sample and for each NOVA category. Mann Whitney *U* tests were performed to determine any significant differences in median HSRs between MP and P products and UP products. The proportion of products within each NOVA category receiving ≥2.5 stars and ≤ 2 stars was calculated.

The rationale for the HSR ranges chosen is that 2.5 stars represents the cut-off at which 50% of ratings fall above and therefore could be considered a ‘healthy pass’. This is relevant because dietary guidelines that incorporate NOVA categories recommend avoidance of UP foods [17, 18]. Also, the ADGs explain that discretionary foods are not an essential part of a healthy diet [49]. Approximately 36% of the energy of Australian adults is derived from discretionary foods (39% for children) [50]. The Australian population needs to consume less discretionary food and to support this behaviour such foods should only be able to display ≤2 ‘health’ stars, i.e. less than half of the maximum 5 stars available, to lessen the risk of them being perceived to be a healthy food. Furthermore, Talati et al. has investigated consumer perceptions of the HSR, with ≥3 stars generally considered healthy and ≤ 2 stars considered unhealthy amongst focus group participants [51].

This sample was previously analysed to determine accordance with the ADGs, coded by the categories visually represented in the 2013 Australian Guide to Healthy Eating: five food group (FFG) foods (fruit; vegetables; grain foods; meat/eggs/tofu/nuts/seeds/legumes; milk/yoghurt/cheese/alternatives; and mixed meals consisting mostly of FFG foods), discretionary foods, and a small number of ‘other’ foods (oils; flour; formulated supplementary foods (FSF); and water). The frequencies and median HSRs of products classified as MP, PCI, P, and UP within each of the ADGs food categories (using data from previous research [35]) were calculated. In addition, the frequencies of each Mintel food category and sub-category allocated to each processing level were produced to examine product characteristics (see Additional file 1: Table S1). A Chi Square test was performed on the frequency of products allocated to each NOVA category within FFG and discretionary groups to test for associations between ADG grouping and level of processing. Further Mann Whitney *U* tests were performed to determine any significant differences in the medians of each NOVA category within each ADG food group.

## Results

The majority of products in the sample were classified as UP (74.4%), followed by MP (12.5%), and P (11.6%) (Table 1). Products classified as MP, P and UP, all had high median HSRs (4.5, 4 and 3.5, respectively), with statistically significant differences detected between all categories (Mann Whitney *U* test, all *p* < 0.05). A relatively lower median HSR of 1 was observed for PCI products. The HSRs ranged from 0.5–5 stars for both MP and UP products (the most and least processed categories), although the variability was higher for UP products (IQR 1.5) than MP products (IQR 1). The majority of MP (98.1%), P (89.8%) and UP products (76.9%) displayed an HSR ≥ 2.5 stars. The majority of PCI products scored an HSR ≤ 2 stars (89.5%).

**Table 1:** Food product frequency, Health Star Rating (HSR): median; range; IQR; and distributions for NOVA categories

NOVA Category	*n*	%	HSR Median	HSR Range	IQR	*n* HSR ≤ 2 (%)*	*n* HSR ≥ 2.5 (%)*
MP	159	12.5	4.5^δ^	0.5–5	1	3(0.02%)	156(98.1%)
PCI	19	1.5	1^δ^	0.5–4	1	17(89.5%)	2(1.1%)
P	147	11.6	4^δ^	0.5–5	1	15(1.2%)	132(89.8%)
UP	944	74.4	3.5^δ^	0.5–5	1.5	218(23.1%)	726(76.9%)
Total Sample	1269	100	3.5^δ^	0.5–5	1.5	253	1016

*n* number of products, *MP* unprocessed and minimally processed, *PCI* processed culinary ingredients, *P* processed, *UP* ultra-processed

(%)* percentage of products within each NOVA category. ^δ^median significantly different to median of all other NOVA categories (Mann Whitney *U* test, *p* < 0.05)

**Fig. 1:**
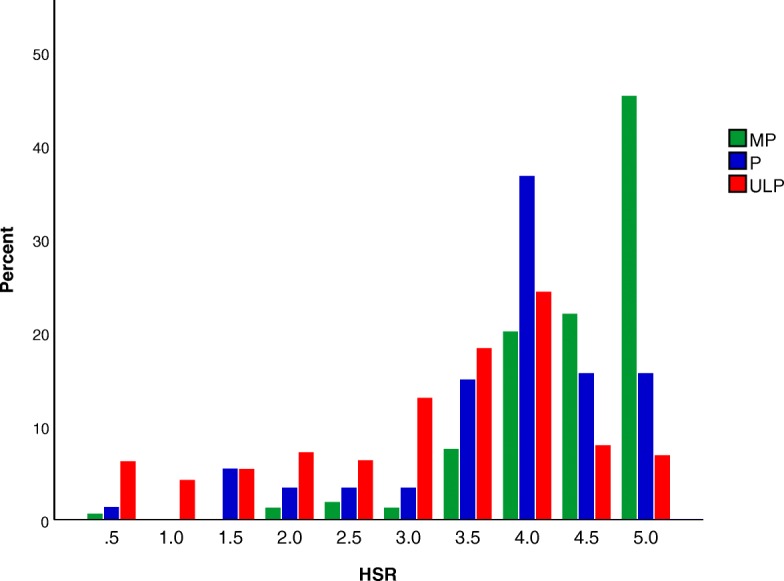
Comparison of the proportion of products displaying Health Star Ratings (HSR) within the minimally processed (MP), processed (P), and ultra-processed (UP) NOVA categories

The HSR distributions of MP, P, and UP products showed all categories skewed towards higher ratings (Fig. 1). MP foods received 5-star ratings more frequently than P and UP products, however a higher proportion of P and UP foods received 4 stars than MP products.

The distribution of NOVA Categories within ADG food groups is shown in Table 2. The majority of both FFG foods and discretionary foods were classified as UP, 61.2 and 94.1%, respectively. There was a significant difference in the level of processing between FFG foods and discretionary foods (Chi Square test, *p* < 0.01), with discretionary foods being classified more often than FFG foods as UP.

**Table 2:** Product frequency and median Health Star Ratings (HSR) of NOVA categories by Australian Dietary Guidelines food group categories

ADG Food Group Categories	MP		PCI		P		UP	
	*n* (%)	HSR	*n* (%)	HSR	*n* (%)	HSR	*n* (%)	HSR
		Med		Med		Med		Med
Five Food Group foods	149(20.6)	4.5^δ^	–	–	132(18.2)	4^δ^	443(61.2)	4
Grains	39	4^δ^	–	–	2	4	151	4
Fruit	45	4.5^δ^	–	–	25	4^δ^	44	5
Vegetables	29	5^δ^	–	–	31	4.5^δ^	45	4
MLNSE	31	5^δ^	–	–	56	4^δ^	61	4
Dairy/Alternatives	5	4	–	–	12	*^δ^	31	4
Mixed Meals	–	–	–	–	6	3.5	111	3.5
Discretionary foods	2(0.4)	**	17(3.2)	1^δ^	12(2.3)	4^δ^	495(94.1)	2.5
Oils	–	–	2	3.5	–	–	–	–
Flour	3	4	–	–	3	4	–	–
FSF	–	–	–	–	–	–	6	4.5
Water	5	5	–	–	–	–	–	–

*MP* unprocessed and minimally processed, *PCI* processed culinary ingredients, *P* processed; *ULP* ultra-processed, *Med* median, *MLNSE* meat, legumes, nuts, seeds and eggs, *FSF* formulated supplementary foods; (%) percentage of products classified in each NOVA category within ADG food group categories

*value = 2.2; **value = 1.25 (due to the number of products in these categories a median HSR could not be calculated)

^δ^median significantly different to median of UP foods within the same ADG group (Mann Whitney *U* test, *p* < 0.05)

The FFG foods classified as MP had a significantly higher median HSR (4.5) to P (4) and UP (4) products (Mann Whitney *U* test, *p* < 0.05). However, in the fruit and discretionary food categories the median HSRs for UP products were higher than the median HSRs for MP products, though the difference was not significant (Mann Whitney *U*-test). The median HSRs for dairy foods were equal for MP and UP products at 4 stars. In the grains category the HSR did not discriminate between levels of processing, with all median HSRs at 4 stars, although medians for MP and UP products were found to be significantly different (Mann Whitney U-test, *p* < 0.05).

## Discussion

The results reveal not only is the HSR being displayed mostly on UP foods but in three-quarters of instances these UP foods were displaying ≥2.5 HSRs. However, a significant difference was observed between the median HSR scores of all new MP, PCI, P, and UP food products sampled indicating the HSR has some power to differentiate between foods in accordance with their level of processing. The public health challenge is that this differentiation power operates as a form of fine control within a system that more fundamentally is calibrated poorly.

The predominant reasons why the majority of new UP foods entering the food marketplace are eligible to display high HSRs are technical weaknesses, a design flaw and governance limitations with the HSR system. The technical weaknesses with the HSR system relate to the algorithm being based on arbitrary cut off levels for scoring selected nutrients and ingredients such as sugar that tend to be higher in UP foods. For example, an UP ice confectionary received 3 health stars despite consisting of only water, sugar, flavouring substances, and preservatives (> 99% of its energy derived from added sugar). There is an underpinning design flaw in the HSR resulting from the system’s reductionist interpretation of nutrition science. Nutrition science explains food and health relationships in terms of the multiple interactions among nutrients, food ingredients and the physical properties of the food itself, as well as how the food is prepared and consumed within the overall dietary pattern [52–54]. Reducing this nutritional complexity of food to a selected number of nutrients abstracted from their food source and without consideration of the food’s broader composition and contexts such as level of processing misrepresents nutrition science. The HSR system’s voluntary governance arrangement also likely contributes to skewing the profile of HSRs displayed on UP foods because it enables manufacturers to display HSRs selectively on foods that are eligible for high HSRs.

FOP labelling is being increasingly investigated around the world and although systems differ slightly, the majority are based on similar nutrient profiling principles to those used in the HSR system. Thus, we expect our results to be broadly generalisable to those FOP labels implemented in other countries. For instance, a recent study analysing all packaged foods available in the Canadian marketplace reflects a similar result, with UP foods significantly more likely than lesser-processed foods to display a symbol or summary system FOP label [55].

Examination of the characteristics of MP, P, and UP foods displaying HSRs revealed that FFG foods spanned all levels of processing, yet, the majority (61.2%) were UP foods. This result may reflect limitations in both the ABS criteria, used to classify FFG and discretionary foods, and/or NOVA criteria in classifying level of food processing. For instance, using NOVA’s food processing criteria, foods considered suitable FFG food options in the ADG recommendations, such as some ready-to-eat meals consisting of vegetables, would be categorised as UP. Therefore, NOVA may lack the nuance necessary to correctly identify certain foods that although processed, are considered suitable selections for a healthy diet. The FFG foods received relatively high HSRs, yet the negligible difference in median HSRs within the FFG foods indicates the HSR system cannot accurately discriminate between MP products and UP products. And although the high median HSRs for FFG foods is a positive outcome, there is still a need to distinguish the more highly processed versions of these products if the HSR label is to be effective in promoting healthy diets.

The inability of the HSR to accurately discriminate against UP foods is particularly apparent when examining HSRs within certain ADG food groups. For example, the median HSR for grain foods was four stars for both MP and UP products. This finding may be in part due to added sugar being inadequately ‘penalised’ by the HSR algorithm [35]. Breakfast cereals comprised 57.9% of the grains category, most of which were classified as UP with HSRs ranging from 2.5–5 stars (see Additional file 1: Table S1). The majority of these products were breakfast cereals with added sugars. Conversely, examples of MP foods were plain oats, dried pasta, and rice, yet these had the same range of HSRs; demonstrating added sugars in cereals have little effect on resulting scores. Similarly, the HSR medians in the sub-categories of fruit and fruit snacks varied minimally over MP, P and UP categories. This may likely be due to manufacturers adding fruit juices and concentrates to products, which then score positively as fruit components [33].

Sixty-six UP products received an HSR ≥ 4 in the database’s sub-category of Snack/Cereal/Energy Bars (see Additional file 1: Table S1). Products within this category included protein bars, muesli bars and energy balls. These products often contained fruit juices, fruit purees, dried fruit, protein powders, nuts, seeds, and processed fibres, all enabling the products to increase their scores while obscuring high sugar and energy contents and highly processed ingredients lists. Although fruit juices are classified within the fruit group in the ADGs [49], many products in the sample were UP, with 29 UP juice products receiving 5 stars (juices were classified as UP if they contained added sugar or industrial additives). This is in comparison to unprocessed whole fruit; for example, a packaged sliced strawberries product received only 4.5 stars. The ADGs state that juice should “only be used occasionally as a substitute for other foods in the [fruit] group”, as juice lacks the fibre found in fresh fruit and is high in naturally occurring sugars [49].

In relation to the HSR’s role in incentivising food reformulation, caution is needed to ensure that nutrition science principles are not compromised. For instance, encouraging modest reductions in the risk nutrient profile of new UP food products entering the marketplace by assigning an increased HSR could risk creating a ‘health aura’ effect, giving the impression that these foods are superior in nutritional value to recommended whole food options [56–58]. This outcome is particularly concerning given the observation of the relatively high frequency of UP food products taking advantage of FOP labelling and reformulation agenda in other countries. A study investigating the Choices label in the Netherlands, found that although the label was encouraging the development of new reformulated products, the majority of these were UP snack foods (including ice-cream, fruit drinks, and liquorice) [59].

### Strengths and limitations

This was the first study to comprehensively analyse all new packaged food products released into the Australian retail food supply displaying the HSR label against food processing criteria. It is relevant that new products in particular were analysed, as this provides insights into the potential risks and benefits of innovations, such as new product development and reformulation, to improve the healthiness of the food supply. This study did not assess previously existing products on the market that have applied the HSR label, the market share of HSR labelled products, or population dietary intake behaviours that may be influenced by the label.

Some misclassification of the level of food processing may have occurred due to the difficulty in matching novel processed food products to corresponding items in the AUSNUT 2011–2013 food composition database, which does not necessarily reflect the evolving nature of the food supply and current consumption trends. O’Halloran et al. also recognised that the initial application of NOVA to the AUSNUT food composition database was not straightforward [23]. However, a documented and transparent method was established to reduce misclassification, with difficult-to-classify products (approximately 5% of the sample) discussed and their classification resolved by consensus among the three researchers. The sample size is modest compared to the proportion of all products displaying the HSR label. However, the sample is an accurate representation of all new packaged food products displaying the label, meeting the aims for this research in analysing the effect of the HSR system on new products released since the system’s implementation.

NOVA is not without its critics as a valid system for assessing the healthiness of food products. For instance, Gibney et al. have challenged the NOVA approach arguing that it is too simple and lacks the ability to contribute to research on overall adequate dietary patterns, or the development of food-based dietary guidelines [60]. However, Monteiro et al. comment, what is “more important than level of complexity is whether the system of categorisation works to predict the nutritional quality of diets and risk of disease” [61], which the evidence confirms and is increasingly supported by studies reporting plausible biological mechanisms [3]. In addition, NOVA is the most widely published system reporting on relationships between food processing and health outcomes [62].

### Future research directions

Future research should aim to assess all products displaying an HSR for their alignment with NOVA categories to confirm these findings apply to the broader food supply. There is also the need to further investigate the relationship between the HSR system, reformulation, and food processing by identifying food products that have increased their HSRs since the system’s implementation and examining the nutrient profile modifications. Additionally, the investigation of a causal relationship between the HSR system and the incentive of food processing activities and the promotion of UP foods will be an important follow up to this research. The increasing production and consumption of UP foods has implications for the security of the food system, extending beyond concerns for population dietary excesses and imbalances. When criteria for sustainable dietary patterns are reflected in a suitable framework it will be important for the HSR system to be assessed for its alignment with such a scheme. Lastly, continued monitoring of the HSR system, or any future iteration, for its relationship to level of processing in Australia will be needed, as well as a similar analysis on FOP labels implemented in other countries.

### Improving the current HSR system to account for ultra-processed foods

The HSR system was not explicitly designed to account for UP foods in the calculation of star ratings. But it was intended to protect public health. Into the future, the system could be reformed to not only encourage the consumption of predominantly MP and P foods, but also help consumers identify UP food products as items for which consumption should be discouraged.

Two reforms to technical aspects of the HSR algorithm could help improve the HSR system’s alignment with NOVA criteria. Firstly, the algorithm currently calculates baseline points based on total sugar, with both added sugars and intrinsic sugars contributing to a negative weighting. One study found that including added sugar instead of total sugar in the algorithm results in better discrimination between FFG foods and discretionary foods [63]. A greater penalty for added sugar is likely to also result in lower star ratings for UP foods, considering high amounts of added sugar are present in many UP foods and non-existent in unprocessed foods. Secondly, modifying points allocated for protein, fibre, or FVNL content, should not be calculated when added in the form of processed ingredients, such as soy isolates, inulin and fruit concentrates, thereby preventing UP foods from masking poor nutrient profiles with ‘positive’ additions to increase their HSRs.

There is a significant design distinction between nutrient profiling and food processing profiling systems. However, reform to FOP labels informed by nutrient profiling involving the application of a level of processing criteria could help lessen the contrasting outputs from the two approaches. For instance, the HSR system should determine that UP foods are ineligible to display the positively-orientated HSR star graphic and instead investigate the use of warning symbols.

If these technical and design reforms to the current HSR system can be achieved, the system’s implementation should then be mandated. A mandatory implementation would prevent food manufacturers from selectively displaying HSRs on those products qualifying for relatively high HSRs.

## Conclusions

Ultra-processed foods are over-represented in new packaged food products engaging with the HSR system and the majority are displaying relatively high HSRs. The nutrient profiling approach, with its limited selection of nutrients and arbitrary cut-off levels, is largely unable to represent the relationship between food processing and health outcomes. In principle, a FOP label could provide an important function in alerting consumers to UP foods and help counter misleading health marketing of these products, however the HSR in its current form is misrepresenting the healthiness of new food products and creating a risk to nutrition behaviour.

Into the future, there will need to be close scrutiny of the impact of the coupling of the HSR system and food reformulation if the evidence on food processing and health is not to be compromised. Using the prospect of garnering a higher number of HSRs as an incentive to drive the reformulation of UP foods may help modify the levels of certain nutrients in that food, but it can’t disguise the fact that the food remains a UP food and its consumption needs to be discouraged in the first place.

## Additional files

Additional file 1: **Table S1.** Frequency and median of unprocessed and minimally processed foods, processed foods, and ultra-processed foods divided by food groups based on the Australian Dietary Guidelines, and Mintel category and sub-category. (DOCX 59 kb)

## Abbreviations

ADGs: Australian Dietary Guidelines
FFG: Five Food Group
FOP: Front-of-pack
FVNL: Fruit, vegetable, nut and legume
GNPD: Global New Product Database
HSR: Health star rating
MP: Unprocessed and minimally processed
P: Processed
PCI: Processed culinary ingredients
UP: Ultra-processed

## References

[1] Institute for Health Metrics and Evaluation: Australia. http://www.healthdata.org/australia. Accessed 3 Oct 2017.

[2] Monteiro CA, Cannon G, Moubarac J-C, Levy RB, Louzada MLC, Jaime PC. The UN decade of nutrition, the NOVA food classification and the trouble with ultra-processing. Public Health Nutr. 2017. 10.1017/S1368980017000234. 10.1017/S1368980017000234 PMC10261019 28322183

[3] Zinöcker KM, Lindseth AI. The Western diet–microbiome-host interaction and its role in metabolic disease. Nutrients. 2018;10. 10.3390/nu10030365 PMC5872783 29562591

[4] Moubarac J-C, Batal M, Louzada ML, Steele EM, Monteiro CA. Consumption of ultra-processed foods predicts diet quality in Canada. Appetite. 2017. 10.1016/j.appet.2016.11.006:512. 10.1016/j.appet.2016.11.006 27825941

[5] Louzada ML, Martins APB, Canella DS, Baraldi LG, Levy RB, Claro RM, Moubarac J-C, Cannon G, Monteiro CA (2015). Ultra-processed foods and the nutritional dietary profile in Brazil. Rev Saude Publica.

[6] Cornwell B, Villamor E, Baylin A, Mora-Plazas M, Marin C, Monteiro CA (2018). Processed and ultra-processed foods are associated with lower-quality nutrient profiles in children from Colombia. Public Health Nutr.

[7] Martinez Steele E, Popkin BM, Swinburn B, Monteiro CA (2017). The share of ultra-processed foods and the overall nutritional quality of diets in the US: evidence from a nationally representative cross-sectional study. Popul Health Metrics.

[8] Slimani N. Contribution of highly industrially processed foods to the nutrient intakes and patterns of middle-aged populations in the European prospective investigation into Cancer and nutrition study. Eur J Clin Nutr. 2009;206. 10.1038/ejcn.2009.82 19888275

[9] Louzada M, Baraldi LG, Steele EM, Martins AP, Canella DS, Moubarac JC, Levy RB, Cannon G, Afshin A, Imamura F, Mozaffarian D, Monteiro CA. Consumption of ultra-processed foods and obesity in Brazilian adolescents and adults. Prev Med. 2015. 10.1016/j.ypmed.2015.07.018:9. 10.1016/j.ypmed.2015.07.018 26231112

[10] Tavares LF, Fonseca SC, Garcia Rosa ML, Yokoo EM (2012). Relationship between ultra-processed foods and metabolic syndrome in adolescents from a Brazilian family doctor program. Public Health Nutr.

[11] Rauber F, Campagnolo PDB, Hoffman DJ, Vitolo MR (2015). Consumption of ultra-processed food products and its effects on children’s lipid profiles: a longitudinal study. Nutr Metab Cardiovasc Dis.

[12] De Deus Mendonça R, Pimenta AM, Gea A, Martinez-Gonzalez MA, Bes-Rastrollo M, Souza Lopes AC (2017). Ultra-processed food consumption and the incidence of hypertension in a mediterranean cohort: the seguimiento universidad de Navarra project. Am J Hypertens.

[13] Fiolet T, Srour B, Sellem L, Kesse-Guyot E, Allès B, Méjean C, Deschasaux M, Fassier P, Latino-Martel P, Beslay M (2018). Consumption of ultra-processed foods and cancer risk: results from NutriNet-Santé prospective cohort. BMJ.

[14] von Koerber K, Bader N, Leitzmann C (2017). Wholesome nutrition: an example for a sustainable diet. Proc Nutr Soc.

[15] Niles MT, Ahuja R, Barker T, Esquivel J, Gutterman S, Heller MC, Mango N, Portner D, Raimond R, Tirado C, Vermeulen S. Climate change mitigation beyond agriculture: a review of food system opportunities and implications. Renew Agric Food Syst. 2018. 10.1017/S1742170518000029:1-12.

[16] Vandevijvere S, Monteiro C, Krebs-Smith SM, Lee A, Swinburn B, Kelly B, Neal B, Snowdon W, Sacks G (2013). Monitoring and benchmarking population diet quality globally: a step-wise approach. Obes Rev.

[17] Ministry of Health of Brazil. Dietary guidelines for the Brazilian population: Ministry of Health of Brazil; 2014. http://www.fao.org/nutrition/education/food-dietary-guidelines/regions/countries/brazil/en/. Accessed 21 May 2017

[18] Food and Agricultural Organization of the United Nations. Food-based dietary guidelines - Uruguay. http://www.fao.org/nutrition/education/food-dietary-guidelines/regions/countries/uruguay/en/. Accessed 21 May 2017 2017.

[19] Martins APB, Levy RB, Claro RM, Moubarac JC, Monteiro CA (2013). Increased contribution of ultra-processed food products in the Brazilian diet (1987-2009). Rev Saude Publica.

[20] Juul F, Hemmingsson E (2015). Trends in consumption of ultra-processed foods and obesity in Sweden between 1960 and 2010. Public Health Nutr.

[21] Solberg SL, Terragni L, Granheim SI (2015). Ultra-processed food purchases in Norway: a quantitative study on a representative sample of food retailers. Public Health Nutr.

[22] Venn D, Banwell C, Dixon J (2017). Australia’s evolving food practices: a risky mix of continuity and change. Public Health Nutr.

[23] O’Halloran SA, Lacy KE, Grimes CA, Woods J, Campbell KJ, Nowson CA. A novel processed food classification system applied to Australian food composition databases. J Hum Nutr Diet. 2017. 10.1111/jhn.12445. 10.1111/jhn.12445 28124481

[24] Pulker CE, Scott JA, Pollard CM. Ultra-processed family foods in Australia: nutrition claims, health claims and marketing techniques. Public Health Nutr. 2017. 10.1017/S1368980017001148:1-11. 10.1017/S1368980017001148 PMC5729842 28714433

[25] Spiteri SA, Olstad DL, Woods JL. Nutritional quality of new food products released into the Australian retail food market in 2015 - is the food industry part of the solution? BMC Public Health. 2018;18, 222. 10.1186/s12889-018-5127-0 PMC5804078 29415698

[26] Luiten CM, Steenhuis IH, Eyles H, Ni Mhurchu C, Waterlander WE (2016). Ultra-processed foods have the worst nutrient profile, yet they are the most available packaged products in a sample of New Zealand supermarkets. Public Health Nutr.

[27] The World Health Organization. Report of the the Commission on Ending Childhood Obesity: WHO; 2016. http://www.who.int/end-childhood-obesity/publications/echo-report/en/. Accessed 06 Sep 2018

[28] Codex Alimentarius Commission. Discussion paper on consideration of issues regarding front-of-pack nutrition labels. 2017, FAO. https://www.google.com/url?q=http://www.fao.org/fao-who-codexalimentarius/sh-proxy/en/%3Flnk%3D1%26url%3Dhttps%25253A%25252F%25252Fworkspace.fao.org%25252Fsites%25252Fcodex%25252FMeetings%25252FCX-714-44%25252FWD%25252Ffl44_03_%252Badd%252B1e.pdf&sa=U&ved=0ahUKEwjHjIjOrcHdAhVB5rwKHSmABCYQFggFMAA&client=internal-uds-cse&cx=018170620143701104933:i-zresgmxec&usg=AOvVaw2cC8U3rgnqS8UVzngIy4DV. Accessed 17 Sept 2018.

[29] Blewett NGN, Pettigrew S, Reynolds C, Yeatman H (2011). Labelling logic: review of food labelling law and policy.

[30] Department of Health: About Health Star Ratings**.** http://healthstarrating.gov.au/internet/healthstarrating/publishing.nsf/Content/About-health-stars. Accessed 2 May 2017.

[31] Department of Health: About the partnership. http://www.health.gov.au/internet/main/publishing.nsf/Content/about-the-partnership. Accessed 7 Aug 2017.

[32] Department of Health: Health Star Rating campaign. http://healthstarrating.gov.au/internet/healthstarrating/publishing.nsf/Content/health-star-rating-campaign. Accessed 14 Jan 2018.

[33] Food Standards Australia New Zealand: Guide for industry to the Health Star Rating Calculator: version 5. 2016, http://healthstarrating.gov.au/internet/healthstarrating/publishing.nsf/Content/guide-for-industry-document. Accessed 21 Apr 2018.

[34] Food Regulation Secretariat: Australia and New Zealand Ministerial Forum on Food Regulation Communique 28. 2017**.** http://foodregulation.gov.au/internet/fr/publishing.nsf/content/forum-communique-2017-April. Accessed 13 Sept 2017.

[35] Lawrence M, Dickie S, Woods J (2018). Do nutrient-based front-of-pack labelling schemes support or undermine food-based dietary guideline recommendations? Lessons from the Australian health star rating system. Nutrients.

[36] Jones A, Rådholm K, Neal B (2018). Defining ‘unhealthy’: a systematic analysis of alignment between the Australian dietary guidelines and the health star rating system. Nutrients.

[37] National Health and Medical Research Council. Eat for Health: Australian Dietary Guidelines Summary. 2013, NHMRC. https://www.eatforhealth.gov.au/sites/default/files/content/The%20Guidelines/n55a_australian_dietary_guidelines_summary_131014_1.pdf. Accessed 23 Apr 2018.

[38] Dunford E, Cobcroft M, Thomas M, Wu J. Technical report: alignment of NSW Healthy Food Provision Policy with the Health Star Rating system: NSW Ministry of Health; 2015. http://www.health.nsw.gov.au/heal/Publications/health-star-rating-system.pdf. Accessed 26 Feb 2018

[39] Crino M, Sacks G, Dunford E, Trieu K, Webster J, Vandevijvere S, Swinburn B, Wu J, Neal B. Measuring the healthiness of the packaged food supply in Australia. Nutrients. 2018;10. 10.3390/nu10060702 PMC6024847 29857517

[40] Jones A, Dunford E, Crossley R, Thout RS, Rayner M, Neal B. An evaluation of the healthiness of the Indian packaged food and beverage supply. Nutrients. 2017;9. 10.3390/nu9101103 PMC5691719 28991201

[41] Lawrence M, Woods J. Re: Jones et al., Nutrients 2018, 10, 501. Nutrients. 2018;10. 10.3390/nu10060746 PMC6024755 29890721

[42] Food Regulation Secretariat. Front-of-pack labelling committee and working group meetings. http://foodregulation.gov.au/internet/fr/publishing.nsf/Content/frontofpackcommittee. Accessed 16 Aug 2017.

[43] Cooper SL, Pelly FE, Lowe JB. Assessment of the construct validity of the Australian health star rating: a nutrient profiling diagnostic accuracy study. Eur J Clin Nutr. 2017. 10.1038/ejcn.2017.23:1-7. 10.1038/ejcn.2017.23 28294168

[44] Food Regulation Secretariat: Front-of-pack labelling update. http://foodregulation.gov.au/internet/fr/publishing.nsf/Content/front-of-pack-labelling-1#u040612. Accessed 26 Apr 2017.

[45] Food Standards Australia New Zealand. About AUSNUT 2011–13. http://www.foodstandards.gov.au/science/monitoringnutrients/ausnut/Pages/about.aspx. Accessed 22 Aug 2017.

[46] Monteiro CA, Cannon G, Moubarac J-C, Levy RB, Louzada MLC, Jaime PC (2017). The UN Decade of Nutrition, the NOVA food classification and the trouble with ultra-processing. Public Health Nutr.

[47] Dunford E, Webster J, Woodward M, Czernichow S, Lun Yuan W, Jenner K, Ni Mhurchu C, Jacobson M, Campbell N, Neal B (2012). The variability of reported salt levels in fast foods across six countries: opportunities for salt reductions. Can Med Assoc J.

[48] The Food Monitoring Group (2012). International collaborative project to compare and track the nutritional composition of fast foods. BMC Public Health.

[49] National Health and Medical Research Council: Australian Dietary Guidelines. 2013. https://www.eatforhealth.gov.au/sites/default/files/content/n55_australian_dietary_guidelines.pdf. Accessed 26 Feb 2018.

[50] Australian Bureau of Statistics: Australian health survey: nutrition first results - foods and nutrients, 2011-12. Cat 4364055007 http://www.abs.gov.au/ausstats/abs@.nsf/Lookup/by%20Subject/4364.0.55.007~2011-12~Main%20Features~Discretionary%20foods~700. Accessed 19 Apr 2017.

[51] Talati Z, Pettigrew S, Kelly B, Ball K, Dixon H, Shilton T (2016). Consumers’ responses to front-of-pack labels that vary by interpretive content. Appetite.

[52] Scrinis G (2013). Nutritionism : the science and politics of dietary advice.

[53] Hjerpsted J, Leedo E, Tholstrup T. Cheese intake in large amounts lowers LDL-cholesterol concentrations compared with butter intake of equal fat content: American Society for Clinical Nutrition, Inc; 2011. p. 1479. 10.3945/ajcn.111.022426 22030228

[54] Thorning TK, Bertram HC, Bonjour J-P, de Groot L, Dupont D, Feeney E, Ipsen R, Lecerf JM, Mackie A, McKinley MC (2017). Whole dairy matrix or single nutrients in assessment of health effects: current evidence and knowledge gaps. Am J Clin Nutr.

[55] Christoforou A, Dachner N, Tarasuk V, Mendelson R. Front-of-package nutrition references are positively associated with food processing. Public Health Nutr. 2017. 10.1017/S1368980017001057:1-10. 10.1017/S1368980017001057 PMC10260836 29227216

[56] Scrinis G (2016). Reformulation, fortification and functionalization: big food corporations’ nutritional engineering and marketing strategies. J Peasant Stud.

[57] Bellatti A, Simon M (2011). Regulating front of package labeling: an exercise in futility?. J Hunger Environ Nutr.

[58] Nestle M (2018). Public health implications of front-of-package labels. Am J Public Health.

[59] Vyth EL, Steenhuis IHM, Roodenburg AJC, Brug J, Seidell JC (2010). Front-of-pack nutrition label stimulates healthier product development: a quantitative analysis. Int J Behav Nutr Phys Act.

[60] Gibney MJ, Forde CG, Mullally D, Gibney ER. Ultra-processed foods in human health: a critical appraisal: American Society for Clinical Nutrition, Inc; 2017. p. 717. 10.3945/ajcn.117.160440. 10.3945/ajcn.117.160440 28793996

[61] Monteiro CA, Cannon G, Moubarac J-C, Levy RB, Louzada MLC, Jaime PC (2017). Ultra-processing. An odd ‘appraisal’. Public Health Nutr.

[62] Kelly B, Jacoby E (2018). Public health nutrition special issue on ultra-processed foods. Public Health Nutr.

[63] Peters SAE, Dunford E, Jones A, Ni Mhurchu C, Crino M, Taylor F, Woodward M, Neal B. Incorporating added sugar improves the performance of the health star rating front-of-pack labelling system in Australia. Nutrients. 2017;9. 10.3390/nu9070701 PMC5537816 28678187

