# Supplementary material for: Analysing the use of the Australian Health Star Rating system by level of food processing
Source: Int J Behav Nutr Phys Act. 2018 Dec 13;15:128. doi: 10.1186/s12966-018-0760-7 (PMC6293654; doi:10.1186/s12966-018-0760-7)
Supplement: Supplementary file 1 — Table S1. Frequency and median of unprocessed and minimally processed foods, processed foods, and ultra-processed foods divided by food groups based on the Australian Dietary Guidelines, and Mintel category and sub-category. (DOCX 59 kb) [file 12966_2018_760_MOESM1_ESM.docx]

**Table S1.** Frequency and median of unprocessed and minimally processed foods, processed foods, and ultra-processed foods divided by food groups based on the Australian Dietary Guidelines, and Mintel category and sub-category.

|  | **MP** | | | **P** | | | **UP** | | |
| --- | --- | --- | --- | --- | --- | --- | --- | --- | --- |
| **ADG Group** |  | ***n***  **(%)** | **HSR**  **Median** |  | ***n***  **(%)** | **HSR**  **Median** |  | ***n***  **(%)** | **HSR**  **Median** |
| **FFG** | All Categories | 149  (20.6) | 4.5 | All Categories | 132  (18.2) | 4 | All Categories | 443  (61.2) | 4 |
| **Grains** | All Categories | 39 | 4 |  | 2 | 4 |  | 151 | 4 |
|  | Bakery | 1 | 5 | Side Dishes  *Stuffing, Polenta & Other* | 2 | 4 | Bakery  *Bread & Bread Products*  *Savoury Biscuits/Crackers* | 22  *6*  *16* | 3.5  *3.75**  *3.5* |
|  | Breakfast Cereals | 8 | 5 |  |  |  | Breakfast Cereals | 105 | 4 |
|  | Side Dishes  *Rice*  *Pasta*  *Stuffing, Polenta & Other* | 30  *8*  *14*  *2* | 4  *3.75**  *4.5*  *4* |  |  |  | Meals  *Instant Noodles*  *Instant Pasta*  *Meal Kits*  *Prepared Meals* | 14  *6*  *5*  *1*  *2* | 3.5  *3*  *3.5*  *4*  *3.5* |
|  |  |  |  |  |  |  | Side Dishes  *Rice*  *Stuffing, Polenta & Other* | 6  *4*  *2* | 3.5  *3.5*  *4* |
|  |  |  |  |  |  |  | Snacks  *Rice Snacks* | 4  *4* | 2  *2* |
| **Fruit** | All Categories | 45 | 4.5 | All Categories | 25 | 4 | All Categories | 44 | 5 |
|  | Desserts & Ice Cream | 1 | 4.5 | Desserts & Ice Cream | 4 | 4 | Desserts & Ice Cream | 2 | 4.25* |
|  | Fruit | 18 | 4.5 | Fruit | 2 | 3.5 | Fruit | 4 | 4 |
|  | Juice | 17 | 5 | Sauces & Seasonings | 1 | 1.5 | Juice | 26 | 5 |
|  | Snacks  *Fruit Snacks*  *Snack Mixes* | 9  *8*  *1* | 4  *4*  *4* | Snacks  *Fruit Snacks*  *Snack Mixes*  *Snack/Cereal/Energy Bars* | 18  *3*  *3*  *12* | 4  *4*  *3.5*  *4* | RTD (Iced Tea) | 1 | 2.5 |
|  |  |  |  |  |  |  | Sauces & Seasonings | 2 | 4.5 |
|  |  |  |  |  |  |  | Snacks  *Fruit Snacks*  *Snack Mixes*  *Snack/Cereal/Energy Bars* | 9  *2*  *1*  *6* | 4  *4.5*  *3*  *4* |
| **Vegetables** | All Categories | 29 | 5 | All Categories | 31 | 4.5 | All Categories | 45 | 4 |
|  | Vegetables | 22 | 5 | Vegetables | 11 | 4.5 | Vegetables | 2 | 4.25* |
|  | Juice Drinks | 2 | 5 | Meals  *Salads* | 1 | 4.5 | Juice Drinks | 2 | 5 |
|  | Meals  *Salads*  *Meal Kits* | 3  *1*  *2* | 4.5  *4.5*  *4.5* | Sauces & Seasonings | 3 | 4 | Meals  *Meal Kits*  *Prepared Meals* | 9  *1*  *8* | 4  *3.5*  *4* |
|  | Side Dishes  *Pasta* | 2 | 5 | Side Dishes  *Potato Products* | 8 | 3.75* | Processed Fish, Meat & Egg Products | 1 | 4.5 |
|  |  |  |  | Snacks  *Vegetable Snacks* | 8 | 5 | Side Dishes  *Potato Products*  *Stuffing, Polenta & Other* | 6  *5*  *1* | 3.5  *3.5*  *3.5* |
|  |  |  |  |  |  |  | Snacks  *Vegetable Snacks* | 1 | 5 |
|  |  |  |  |  |  |  | Soups  *Wet Soup* | 24 | 3.5 |
| **MLNSE** | All Categories | 31 | 5 | All Categories | 56 | 4 | All Categories | 61 | 4 |
|  | Desserts & Ice Cream | 1 | 5 | Vegetables | 6 | 5 | Desserts & Ice Cream | 1 | 3.5 |
|  | Juice Drinks | 4 | 5 | Meals  *Meal Kits*  *Prepared Meals*  *Salads* | 3  *1*  *1*  *1* | 4  *3.5*  *4*  *4.5* | Vegetables | 2 | 4 |
|  | Sauces & Seasonings | 1 | 2.5 | Processed Fish, Meat & Egg Products | 35 | 4 | Meals  *Prepared Meals*  *Salads* | 3  *2*  *1* | 4  *4*  *4.5* |
|  | Snacks  *Nuts*  *Snack Mixes* | 22  *20*  *2* | 5  *5*  *3.5* | Sauces & Seasonings | 1 | 3 | Processed Fish, Meat & Egg Products | 47 | 3.5 |
|  | Sweet Spreads | 3 | 4.5 | Snacks  *Nuts*  *Snack/Cereal/Energy Bars* | 4  *3*  *1* | 5  *5*  *4* | Snacks  *Nuts*  *Snack Mixes*  *Snack/Cereal/Energy Bars* | 7  *4*  *1*  *2* | 4.5  *4*  *5*  *4* |
|  |  |  |  | Sweet Spreads | 7 | 4.5 | Sweet Spreads | 1 | 4 |
| **Dairy** | All Categories | 5 | 4 | All Categories | 12 | 2.2* | All Categories | 31 | 4 |
|  | Dairy  *White Milk* | 5 | 4 | Dairy  *Curd & Quark*  *Hard Cheese*  *Processed Cheese*  *Soft Cheese* | 12  *1*  *6*  *1*  *4* | 2.25*  *1.5*  *2.5*  *2*  *3.25** | Dairy  *Drinking Yoghurt*  *Evaporated Milk*  *Flavoured Milk*  *Plant Based Drinks*  *Processed Cheese*  *Rice/Nut/Grain & Seed Drinks*  *Soy Based Drinks*  *Spoonable Yoghurt* | 27  *1*  *1*  *6*  *4*  *1*  *2*  *2*  *10* | 4.5  *3.5*  *4.5*  *4.5*  *4*  *3.5*  *4*  *4.75**  *5* |
| **Mixed Meals** |  |  |  | All Categories | 6 | 3.5 | All Categories | 111 | 3.5 |
|  |  |  |  | Meals  *Meal Kits*  *Prepared Meals* | 2  *1*  *1* | 3.25*  *3*  *3.5* | Bakery  *Sweet Biscuits* | 1 | 0.5 |
|  |  |  |  | Side Dishes  *Pasta* | 2 | 3.75* | Vegetables | 4 | 5 |
|  |  |  |  | Snacks  *Hors D’oeuvres/Canapés* | 2 | 3.25* | Meals  *Meal Kits*  *Prepared Meals*  *Salads*  *Pastry Dishes*  *Pizzas* | 72  *15*  *50*  *2*  *1*  *4* | 3.5  *3.5*  *3.5*  *4*  *3*  *3* |
|  |  |  |  |  |  |  | Other Beverages  *Meal Replacements* | 1 | 4.5 |
|  |  |  |  |  |  |  | Side Dishes  *Rice* | 1 | 3.5 |
|  |  |  |  |  |  |  | Snacks  *Hors D’oeuvres/Canapes*  *Snack Mixes* | 2  *1*  *1* | 3.5  *3*  *4* |
|  |  |  |  |  |  |  | Soup  *Wet Soup* | 30 | 3.5 |
| **Discretionary** | All Categories | 2  (0.4) | 1.25 |  | 12  (2.3) | 4 |  | 495  (94.1) | 2.5 |
|  | Dairy  *Cream* | 1 | 0.5 | Processed Fish, Meat & Egg Products | 8 | 3.75* | Bakery  *Baking Ingredients & Mixes*  *Bread & Bread Products*  *Cakes, Pastries & Sweet Goods*  *Savoury Biscuits/Crackers*  *Sweet Biscuits* | 107  *23*  *6*  *18*  *11*  *49* | 1.5  *1.5*  *1.75**  *1.5*  *2.5*  *0.5* |
|  | Water | 1 | 2 | Snacks  *Snack/Cereal/Energy Bars* | 4 | 4 | Breakfast Cereals | 11 | 3 |
|  |  |  |  |  |  |  | Carbonated Soft Drinks | 11 | 1 |
|  |  |  |  |  |  |  | Chocolate Confectionary | 18 | 0.5 |
|  |  |  |  |  |  |  | Dairy  *Cream*  *Plant Based Drinks*  *Processed Cheese*  *Sweetened Condensed Milk* | 8  *4*  *2*  *1*  *1* | 1  *0.5*  *1.5*  *1*  *1* |
|  |  |  |  |  |  |  | Desserts & Ice Cream | 45 | 2.5 |
|  |  |  |  |  |  |  | Hot Beverages | 3 | 2 |
|  |  |  |  |  |  |  | Juice Drinks | 5 | 2.5 |
|  |  |  |  |  |  |  | Meals  *Instant Pasta*  *Meal Kits*  *Pastry Dishes*  *Pizzas*  *Prepared Meals* | 24  *1*  *1*  *12*  *5*  *5* | 3  *3*  *3*  *3*  *3*  *3* |
|  |  |  |  |  |  |  | Other Beverages  *Beverage Mixes*  *Meal Replacements* | 4  *3*  *1* | 4.5  *4.5*  *5* |
|  |  |  |  |  |  |  | Processed Fish, Meat & Egg Products | 9 | 3.5 |
|  |  |  |  |  |  |  | RTDs (Iced Tea) | 2 | 2.5 |
|  |  |  |  |  |  |  | Sauces & Seasonings | 48 | 3 |
|  |  |  |  |  |  |  | Savoury Spreads | 12 | 3.75* |
|  |  |  |  |  |  |  | Side Dishes  *Pasta*  *Potato Products* | 6  *1*  *5* | 4.25*  *2*  *4.5* |
|  |  |  |  |  |  |  | Snacks  *Corn-based Snacks*  *Fruit Snacks*  *Hors D’oeuvres/Canapes*  *Popcorn*  *Potato Snacks*  *Snack Mixes*  *Snack/Cereal/Energy Bars*  *Wheat & Other Grain-based Snacks* | 139  *3*  *3*  *18*  *5*  *9*  *1*  *84*  *16* | 3.5  *3.5*  *2.5*  *2*  *4.5*  *2.5*  *2*  *4*  *2.5* |
|  |  |  |  |  |  |  | Soup  *Dry Soup* | 13 | 3.5 |
|  |  |  |  |  |  |  | Sugar & Confectionary | 22 | 2 |
|  |  |  |  |  |  |  | Sweet Spreads | 8 | 0.75* |
| **Flour** | Bakery | 3 | 4 | Bakery | 3 | 4 |  |  |  |
| **FSF** |  |  |  |  |  |  | Dairy | 1 | 4.5 |
|  |  |  |  |  |  |  | Hot Beverages | 1 | 4.5 |
|  |  |  |  |  |  |  | Other Beverages | 4 | 4.5 |
| **Water** | Water | 5 | 5 |  |  |  |  |  |  |

MP=unprocessed and minimally processed; PCI=processed culinary ingredients; P=processed; UP=ultra-processed; ADG=Australian Dietary Guidelines; FFG=five food group; MLNSE=meat, legumes, nuts, seeds and eggs; FSF=formulated supplementary foods; (%) percentage of products classified in each NOVA category within ADG food group categories; *Median could not be calculated.
